# Regulation of osteoclastogenesis and bone resorption by fatty acid metabolism: mechanisms and therapeutic opportunities

**DOI:** 10.3389/fendo.2026.1887006

**Published:** 2026-07-22

**Authors:** Yingying Jin, Chanchan Zheng, Yuyan Xiang, Zichen Zheng, Jianquan Chen

**Affiliations:** Department of Clinical Medicine, School of Medicine, Hangzhou City University, Hangzhou, China

**Keywords:** bone resorption, *de novo* lipogenesis, fatty acid metabolism, fatty acid β-oxidation, osteoclastogenesis, osteolytic diseases, therapeutic targeting

## Abstract

Bone remodeling is orchestrated by osteoclast-mediated resorption and osteoblast-mediated formation. Dysregulated hyperactivity of osteoclasts is a central driver of pathological bone loss in multiple osteolytic diseases, such as postmenopausal osteoporosis, rheumatoid arthritis, and peri-implant osteolysis. Although the core RANKL signaling cascade that governs osteoclast differentiation has been well characterized, accumulating evidence indicates that RANKL also triggers profound metabolic reprogramming during osteoclastogenesis, among which fatty acid (FA) metabolism has emerged as a critical regulator of osteoclast fate and function. In this review, we provide a comprehensive analysis of how FA metabolic pathways regulate osteoclast differentiation, fusion, and function. We first briefly introduce the core FA metabolic pathways relevant to osteoclasts, including FA uptake, FA oxidation, FA storage, *de novo* lipogenesis, and protein S-palmitoylation. We then examine their specific roles in osteoclastogenesis, focusing on the key regulators (namely FATP2, FABP4, CD36, FASN, ACC1, CPT1A, and DGAT1). Furthermore, we evaluate the translational potential of targeting these metabolic nodes for treating osteolytic diseases, and discuss key challenges, including paradoxical stage-dependent functions of FA oxidation, off-target and species-specific toxicities of systemic inhibitors, and the necessity of osteoclast-specific delivery strategies. Finally, we identify critical knowledge gaps and propose future directions. Collectively, targeting osteoclastic FA metabolism offers a promising, yet challenging, therapeutic avenue for osteolytic diseases.

## 1 Introduction

Bone is a dynamic tissue that undergoes continuous renewal through a tightly regulated process known as bone remodeling (1). This lifelong metabolic activity maintains skeletal strength, repairs microdamage, regulates calcium-phosphate homeostasis, and enables adaptive responses to mechanical loading. The remodeling process is orchestrated by two principal cell types: osteoclasts, which resorb bone matrix, and osteoblasts, which subsequently form new bone. These are supported by osteocytes and bone marrow adipocytes, which help coordinate this cellular network. Osteoclasts are large, multinucleated cells derived from the monocyte-macrophage lineage that specialize in degrading mineralized tissue (1, 2). Osteocytes, the most abundant bone cells, originate from osteoblasts and function as key regulators of both osteoclastogenesis and osteoblastogenesis through the secretion of receptor activator of NF-κB ligand (RANKL) and sclerostin, respectively (3). Bone marrow adipocytes, which share a common mesenchymal progenitor with osteoblasts, dynamically regulate the marrow lipid milieu; they can provide fatty acids (FAs) to support osteoblasts during energy stress and also influence osteoclastogenesis through RANKL production (4, 5). Perturbations in osteoclast number or function produce opposing skeletal phenotypes. Insufficient osteoclast activity causes osteopetrosis, characterized by abnormally dense but structurally brittle bone, whereas excessive osteoclast-mediated resorption contributes to pathological bone loss, including osteoporosis, a condition of reduced bone mass and increased fracture risk that represents a major global health burden (2, 6).

Osteoclast differentiation and activation are governed by the master cytokine RANKL, which is produced primarily by mesenchymal lineage cells. RANKL binding to its receptor RANK on osteoclast precursors triggers canonical signaling cascades (including NF-κB, MAPK, and AKT pathways) that converge on the master transcription factor nuclear factor of activated T cells c1 (NFATc1), thereby driving osteoclast gene expression and cell fusion (2, 7). Beyond this well-established signaling framework, recent studies have revealed that RANKL also induces profound metabolic reprogramming during osteoclast differentiation and function (8–14). This metabolic rewiring not only meets energetic and biosynthetic demands, but also generates metabolites that serve as essential substrates for epigenetic modifications, thereby linking cellular metabolism directly to gene expression regulation (13). While RANKL-driven metabolic reprogramming is now recognized as essential for osteoclastogenesis, the specific contributions of distinct metabolic pathways to osteoclast differentiation, multinucleation, and resorptive activity remain incompletely defined (13). Furthermore, whether these metabolic nodes can be therapeutically targeted to selectively modulate osteoclast function without causing systemic metabolic disruption represents a critical translational challenge.

Among the metabolic programs implicated in osteoclast biology, accumulating evidence indicates that FA metabolism critically regulates osteoclast differentiation and activity, both directly through cell-autonomous mechanisms and indirectly via modulation of the bone marrow microenvironment (9, 10, 15–17). This focus is particularly relevant for several reasons. First, FAs are the most abundant energy substrates in the bone marrow niche, which is richly populated by adipocytes that dynamically regulate lipid availability (18, 19). Second, the cellular machinery involved in FA uptake, synthesis, and oxidation is dynamically regulated during osteoclast differentiation (9, 10, 15–17), offering multiple potential therapeutic targets.

In this review, we provide a comprehensive analysis of the experimental evidence linking FA metabolism to osteoclast biology. We begin with an overview of the core pathways of FA metabolism. We then examine how each of these pathways contributes to osteoclast differentiation, fusion, and bone-resorptive function, with particular attention to the specific FA transporters, enzymes, and signaling intermediaries involved. We further discuss the pathophysiological implications of these findings in osteolytic diseases, such as postmenopausal osteoporosis (PMOP), rheumatoid arthritis (RA), and peri-implant osteolysis. Finally, we evaluate the therapeutic potential of targeting FA metabolic nodes in osteoclasts and identify critical gaps in knowledge that warrant future investigation.

## 2 An overview of FA metabolism

FAs are fundamental molecules in living organisms, serving as major energy sources, building blocks for membrane phospholipids and bioactive lipid mediators, and substrates for posttranslational protein modifications. FAs can be classified based on three main structural features: chain length, the presence of branching, and degree of saturation. By chain length, FAs are typically categorized as short-chain (2–6 carbons), medium-chain (8–12 carbons), long-chain (14–18 carbons), or very-long-chain (20 or more carbons) (20). In terms of branching, most FAs are straight-chain, but branched-chain fatty acids (BCFAs) contain methyl groups along the backbone and are commonly found in bacteria and certain animal tissues. Regarding saturation, FAs are divided into saturated (no double bonds), monounsaturated (one double bond), and polyunsaturated (two or more double bonds). These structural differences profoundly influence their physical properties, biological functions, and roles in health and disease.

Cells acquire FAs through two primary routes: uptake of extracellular FAs or endogenous biosynthesis. Once inside the cells, FAs can be oxidized for energy, stored as lipids, or used for protein lipidation (21). A thorough understanding of FA metabolism is essential for elucidating energy homeostasis, cellular physiology, and the pathogenesis of metabolic diseases.

### 2.1 FA transport, binding, and activation

**Figure 1:**
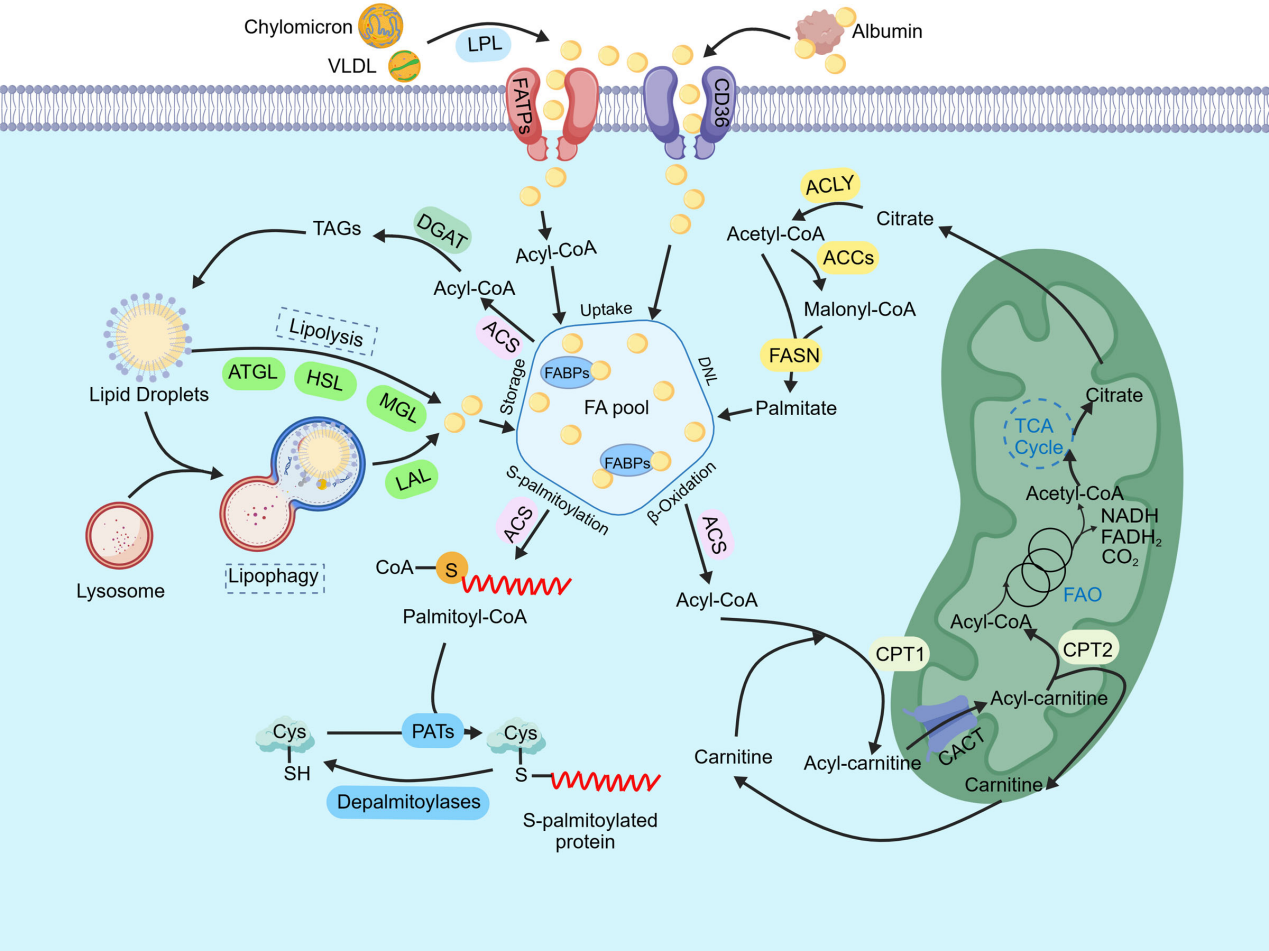
Overview of fatty acid (FA) metabolism. The core pathways of FA metabolism include FA uptake, FA β-oxidation, FA storage, and *de novo* lipogenesis. FA can also be used as a substrate for protein S-palmitoylation.

FA transport addresses the challenge of moving hydrophobic molecules efficiently through aqueous environments. Dietary FAs from the intestine or those released from adipose storage are transported in blood plasma bound to albumin or encapsulated as triglycerides (TAGs) within lipoproteins, such as chylomicrons from diet and very low-density lipoproteins (VLDL) from the liver. At target tissues, FAs dissociate from albumin or are released from lipoproteins by lipoprotein lipase (LPL) (**Figure 1**). While short- and medium-chain FAs can diffuse across the plasma membrane, efficient uptake of long-chain and very-long-chain FAs is mediated by specific transport proteins, primarily CD36 and the fatty acid transport proteins (FATPs, including FATP1–6) (20, 22, 23) (**Figure 1**). Importantly, FATPs also possess intrinsic acyl-CoA synthetase (ACS) activity. Once in the cytoplasm, FAs are bound by cytosolic fatty acid-binding proteins (FABPs, including FABP1–5) (**Figure 1**), which prevents detergent-like membrane damage and facilitates intracellular shuttling. Finally, FAs are activated by one of the 26 mammalian ACS enzymes, which attaches coenzyme A (CoA) to form fatty acyl−CoA (24, 25). This activation step traps the FAs in the cell and prepares it for downstream metabolic pathways.

### 2.2 *De novo* lipogenesis

In addition to exogenous uptake, cells can synthesize FAs from non-lipid precursors, primarily excess dietary carbohydrates such as glucose, and to a lesser extent amino acids or other metabolites, through a process termed *DNL* in the cytosol (26). Acetyl-CoA carboxylase (ACC) catalyzes the initial, rate−limiting step, carboxylating acetyl-CoA to form malonyl-CoA (**Figure 1**). There are two mammalian ACC isoforms, ACC1 and ACC2. ACC1 resides in the cytosol and produces malonyl-CoA primarily for *DNL*, whereas ACC2 is tethered to the outer mitochondrial membrane and generates malonyl-CoA mainly to suppress CPT1 and thereby inhibit fatty acid oxidation (27, 28). Although these isoforms have distinct primary functions, evidence suggests they may perform partially redundant roles under certain conditions (27, 29). Fatty acid synthase (FASN) carries out the subsequent steps of *DNL* (30) (**Figure 1**). This multifunctional homodimeric enzyme uses acetyl-CoA as a primer and repeatedly adds two-carbon units from malonyl-CoA in a cyclic process, ultimately producing palmitate (21). Each elongation cycle consists of four reactions, including condensation, reduction, dehydration, and a second reduction, with NADPH as the reducing agent (21). All reactions are performed by the distinct catalytic domains of FASN (21). As a 16-carbon saturated fatty acid, palmitate can be further elongated by the ELOVL family of elongases and/or desaturated by stearoyl-CoA desaturase 1 (SCD-1) to generate monounsaturated FAs (31, 32).

### 2.3 Fatty acid β-oxidation

FAO is the core catabolic pathway for breaking down FAs to generate ATP. This pathway occurs primarily in the mitochondrial matrix, though very-long-chain FAs undergo initial shortening in peroxisomes before mitochondrial oxidation. To enter mitochondria for β-oxidation, fatty acyl-CoA must traverse the mitochondrial membranes via the carnitine shuttle system. Carnitine palmitoyltransferase I (CPT1) on the outer mitochondrial membrane transfers the acyl group from CoA to carnitine, the resulting acyl-carnitine is transported across the inner membrane by carnitine-acylcarnitine translocase (CACT); and finally carnitine palmitoyltransferase II (CPT2) on the inner membrane transfers the acyl group back to CoA inside the mitochondrion (33) (**Figure 1**). CPT1 serves as a major regulatory point of FAO and is allosterically inhibited by malonyl-CoA, the first committed molecule in fatty acid synthesis (34). This ensures that the cell does not simultaneously burn and build fatty acids. Of note, mammals have three CPT1 genes (*Cpt1a*, *Cpt1b*, and *Cpt1c*), which encode CPT1A, CPT1B, and CPT1C, respectively. These CPT1 proteins may be functionally redundant, although their expression exhibits preferential tissue distribution (34, 35).

Once acyl-CoA is transported into mitochondria, FAO proceeds as a repetitive cyclic process in which 2-carbon units are sequentially cleaved from the carboxyl terminus of activated fatty acyl-CoA (36) (**Figure 1**). Each round comprises four sequential enzymatic reactions: dehydrogenation (acyl-CoA dehydrogenase), hydration (enoyl-CoA hydratase), oxidation (3-hydroxyacyl-CoA dehydrogenase), and thiolysis (thiolase). One cycle yields one acetyl-CoA, one FADH_2_, one NADH, and a shortened fatty acyl-CoA for subsequent rounds. Complete oxidation of a single palmitate molecule yields approximately 106 ATP, far more energy per carbon than glucose oxidation.

### 2.4 Storage of FAs as lipid droplets

Excess FAs are esterified into TAGs and stored in LDs for long-term energy reserves (**Figure 1**). FAs are activated to acyl-CoA and sequentially esterified with glycerol-3-phosphate (derived from glycolysis or glyceroneogenesis) by three enzymes, namely glycerol-3-phosphate acyltransferase (GPAT), acylglycerolphosphate acyltransferase (AGPAT), and diacylglycerol acyltransferase (DGAT) (37–39). DGAT is the rate-limiting enzyme and has two isoforms (DGAT1 and DGAT2). Newly synthesized TAG is then packaged into LDs surrounded by specific proteins such as perilipins (37–39). During energy demand, LD-resident lipases, including adipose triglyceride lipase (ATGL), hormone-sensitive lipase (HSL), and monoacylglycerol lipase (MGL), sequentially break down stored TAGs via lipolysis, with ATGL as the rate-limiting enzyme. Alternatively, FAs can be released from LDs via lipophagy, a lysosomal process mediated by lysosomal acid lipase (LAL) (39).

### 2.5 FAs as substrates for posttranslational lipid modification

Beyond their canonical roles in energy provision and storage, FAs, particularly palmitate (C16:0), serve as essential covalent modifiers of proteins in the process of protein lipidation (40, 41). Among these modifications, S-palmitoylation is the most extensively studied. This post-translational modification involves the reversible attachment of a palmitate moiety to a cysteine residue of a target protein via a labile thioester bond (42). Unlike other lipid modifications such as myristoylation or prenylation, S-palmitoylation is uniquely dynamic and is enzymatically regulated by two opposing families of enzymes (40, 41, 43). Palmitoyl acyltransferases (PATs), which contain a conserved Asp-His-His-Cys (DHHC) motif and catalyze the addition of palmitate (40, 41), whereas depalmitoylases mediate its removal (40, 41) (**Figure 1**). This reversible cycle enables palmitoylation to function as a molecular switch, rapidly controlling protein stability, subcellular trafficking, membrane association, and protein-protein interactions (40, 41, 43).

In summary, FA metabolism is a dynamic, tightly regulated system (**Figure 1**). *DNL* builds FAs when energy is abundant; transport systems deliver exogenous FAs to their destinations; FAO breaks them down efficiently when energy is needed; TAG storage buffers FA toxicity while providing long-term energy reserves; and palmitate availability regulates protein S-palmitoylation. The balance among these interconnected pathways is essential for metabolic health, and dysregulation in any part of this system contributes significantly to prevalent metabolic diseases.

## 3 The role and mechanism of FA metabolism in osteoclastogenesis

Different FA species have shown differential effects on osteoclastogenesis (44). For instance, saturated FAs (SFAs), such as lauric acid (C12) and palmitate (C16), enhanced RANKL-induced osteoclastogenesis. Palmitate can even induce osteoclast formation in the absence of RANKL (45). In addition, SFAs have been reported to promote osteoclast survival (46). In contrast, palmitoleic acid (PLA), a 16-carbon monounsaturated FA, not only suppressed RANKL-induced formation of mature osteoclasts from RAW264.7 cells but also induced apoptosis of these cells (47). Similarly, long-chain and very-long-chain polyunsaturated FAs (PUFAs), particularly n-3 PUFAs such as docosahexaenoic acid (DHA) and eicosapentaenoic acid (EPA), exert negative effects on osteoclastogenesis (44, 48). Furthermore, short-chain FAs (SCFAs), which can be produced by gut microbiota, have been shown to suppress osteoclastogenesis both *in vitro* and *in vivo* (49). Beyond these phenomenological observations, recent studies have begun to unravel the specific FA metabolic pathways that potentially mediate these differential effects (**Table 1**).

**Table 1:** Summary of major FA metabolic pathways and their regulatory effects on osteoclastogenesis.

Metabolic pathway	Key enzymes/regulators	Net effect on osteoclastogenesis	Primary mechanism(s)	Key functional outcome
FA Uptake & Binding	FATP2 FABP4 CD36	Promoting	FATP2: Fuels FAO to generate ATP and ROS, activating NF-κB. FABP4: Elevates intracellular Ca^2+^, activating Calcineurin/NFATc1 axis. CD36: Mediates TSP-1 signaling (PPARγ-c-FOS cascade).	Provides metabolic fuel and amplifies RANKL signaling for precursor commitment.
DNL	FASN ACC1/2	Promoting (Direct)	Provides palmitate for STAT3 S-palmitoylation, driving c-Fos/NFATc1. Upregulates antioxidant enzymes to curb ROS. Activates MAPK signaling.	Supplies essential lipids for S-palmitoylation and signaling.
FAO	CPT1A CPT2 PRMT6	Stage-Dependent (Dual)	Early stage: PRMT6 suppresses FAO to allow glycolysis/HIF-1α switch. Late stage/Fusion: CPT1A drives ATP production and C/EBPβ-clathrin axis for fusion. Sex-dependent (females more affected).	Provides ATP for late-stage fusion/maturation, but must be downregulated early to permit glycolytic reprogramming.
FA Storage (TAG synthesis)	DGAT1	Suppressing	Sequesters pro-inflammatory SFAs (e.g., palmitate) into inert lipid droplets. Limits TNFα production and reduces RANK expression.	Detoxifies lipotoxic FAs, preventing uncontrolled inflammation-driven osteoclastogenesis.

### 3.1 FA uptake and binding in osteoclastogenesis

A recent study identified FATP2-mediated FA transport as a metabolic checkpoint that couples lipid availability to osteoclastogenesis (22). FATP2, which functions both as a transporter for long-chain/very-long-chain FAs and as an ACS, is selectively up-regulated during RANKL-stimulated osteoclastogenesis of bone-marrow–derived macrophages (BMMs). In contrast, expression of other FATPs (FATP1,3,5) and the alternative FA transporter CD36 remains unchanged, while FATP4 and FATP6 are specifically suppressed by RANKL stimulation. Knockdown of FATP2 via *siRNA* or specific inhibition of its transporter activity with the small-molecule Lipofermata suppresses RANKL-induced osteoclastogenesis upstream of NFATc1 and c-Fos, without causing overt toxicity to osteoblasts or impairing their mineralization capacity. Consistently, FATP2 expression is elevated in bone tissues and BMMs from mice fed a high-fat diet (HFD) compared to chow-fed controls, and Lipofermata attenuates HFD-induced osteoclastogenesis *in vitro*. Mechanistically, FATP2 blockade reduces uptake of long-chain and very-long-chain FAs, downregulates key factors involved in FAO, the tricarboxylic acid (TCA) cycle, and oxidative phosphorylation, and consequently lowers intracellular ATP levels. In addition, FATP2 inhibition decreases reactive oxygen species (ROS) levels and thereby attenuates NF-κB signaling. Consistent with these *in vitro* findings, systemic administration of Lipofermata protects against ovariectomy- or lipopolysaccharide (LPS)-induced osteoclastogenesis and bone loss in mice. Thus, FATP2 acts as a metabolic gatekeeper linking FA availability to the ATP and ROS production required for osteoclastogenesis, with its inhibition representing a promising therapeutic strategy for lipid overload-, estrogen deficiency-, or inflammation-driven osteolytic bone loss. However, genetic validation (e.g., conditional *Fatp2* knockout in osteoclast precursors) and clarification of its ACS activity’s role in osteoclast formation remain needed. Notably, the downregulation of FATP4 and FATP6 during osteoclastogenesis raises the possibility that these transporters may have functions distinct from FATP2, potentially serving as negative regulators of osteoclast differentiation. Whether their suppression is a permissive or instructive event that facilitates differentiation, or is merely a consequence of lineage commitment remains to be determined. Future gain- or loss-of-function studies are warranted to elucidate the functional significance of FATP4 and FATP6 downregulation in osteoclastogenesis.

Previous studies have reported conflicting findings regarding CD36’s role in osteoclast formation. Helming et al. found that *Cd36* deletion in murine BMMs did not affect osteoclastogenesis, suggesting it is dispensable for basal osteoclastogenesis (50). In contrast, Koduru et al. showed that silencing *Cd36* in human peripheral blood monocytes partially impairs *in vitro* osteoclastogenesis, and *Cd36* knockout mice exhibit increased spinal trabecular bone mass (51). Further evidence positions CD36 as a key mediator of thrombospondin-1 (TSP-1) signaling (51–53) in osteoclastogenesis. Specifically, TSP-1 binds CD36 on osteoclast precursors to suppress nitric oxide production, thereby promoting osteoclast differentiation and bone resorption (51). This pathway is particularly relevant in pathological conditions such as type 2 diabetes, where neutrophil-derived TSP-1 activates CD36 on macrophages, triggering a PPARγ-POU2F2-c-FOS cascade that enhances osteoclast activity and drives bone loss (53). Thus, although it remains controversial whether CD36 is universally required for basal osteoclastogenesis, it plays a critical role in TSP-1-driven osteoclast differentiation and function, particularly in pathological contexts.

Fatty acid-binding protein 4 (FABP4) is involved in the transport and binding of FAs. Recent work shows that FABP4 amplifies RANKL signaling by elevating intracellular Ca^2+^ and thereby activating the Ca^2+^/calcineurin/NFATc1 transcriptional axis, the key driver of osteoclastogenesis (17). Clinically, serum FABP4 levels are increased in PMOP patients and inversely correlated with bone mineral density (BMD) in these patients (17). *In vitro* experiments demonstrate that recombinant FABP4 or free-fatty-acid mixtures that up-regulate endogenous FABP4 accelerate RANKL-induced osteoclast differentiation and function, whereas genetic disruption of FABP4 or pharmacological inhibition via its selective inhibitor BMS309403 suppresses osteoclast formation (17, 54). Moreover, exogenous FABP4 promote osteoclast activity in the bone marrow of collagen-induced arthritic mice (55). In contrast, FABP4 manipulation has no effect on osteogenic differentiation of bone-marrow mesenchymal stem cells, confirming an osteoclast-specific mechanism (17). Consistent with these results, genetic deletion of *Fabp4* attenuated OVX-induced osteoclastogenesis and bone loss in mice (54). Similarly, oral administration of BMS309403 improves BMD in ovariectomized mice, though it is less potent than alendronate (17). Furthermore, bone-targeted delivery of BMS309403 via bisphosphonate-conjugated nanoparticles enhances drug accumulation in the bone marrow, restoring trabecular structure and BMD to levels comparable to alendronate treatment (17). Collectively, these findings suggest FABP4 as a therapeutic target for PMOP and metabolic bone disorders, with nanoparticle-based delivery offering a strategy to mitigate systemic effects while maximizing bone-specific efficacy.

### 3.2 *DNL* in osteoclastogenesis

In addition to cellular uptake, FA can also be produced by cells via *DNL*. Bermeo et al. demonstrated that inhibiting *DNL* with cerulenin (an inhibitor of FASN targeting its β-ketoacyl synthase activity) significantly attenuated bone loss in ovariectomized mice by increasing bone formation and reducing bone resorption (16). This anti-osteoclastogenic effect was not due to a direct action of cerulenin on osteoclasts, as *in vitro* assays showed no impact on osteoclast differentiation, survival, or resorptive function. Instead, the mechanism involves a reduction in bone marrow adiposity and the secretion of lipotoxic factors, which in turn downregulates the expression of RANKL in the bone marrow microenvironment. Since RANKL is a key mediator of osteoclast formation and activity, its decreased expression disrupts the communication between osteoblasts and osteoclasts, leading to uncoupled bone remodeling and attenuated bone loss. Thus, FASN-driven lipogenesis can enhance osteoclastogenesis through its role in expanding marrow fat and elevating RANKL levels. However, Tang et al. showed that FASN inhibitor C75 stimulates bone mass by increasing bone formation, rather than by inhibiting osteoclastogenesis and bone resorption, in healthy mice (56). In contrast to the above studies, we recently demonstrated that FASN-mediated *DNL* plays a direct role in promoting osteoclastogenesis (10). During RANKL-induced osteoclast differentiation, both *Fasn* expression and *DNL* activity are upregulated. Inhibition of *Fasn*, either through *shRNA* knockdown or pharmacological inhibitors (ASC40 and C75), significantly impairs osteoclast formation. Mechanistically, *Fasn* inhibition deprives the cell of palmitate, blunting palmitoylation-dependent STAT3 activation, which in turn suppresses the expression of c-Fos and NFATc1, the master transcription factors of the osteoclast differentiation. Concomitantly, FASN blockade counteracts intracellular ROS accumulation by upregulating antioxidant enzymes and attenuates RANKL-induced MAPK signaling. Targeting this pathway with ASC40 protects mice from ovariectomy- and titanium-nanoparticle–induced osteoclast formation and bone loss. These findings establish *DNL* as a novel metabolic regulator of osteoclast differentiation through palmitate supply and validate its potential as a therapeutic target in osteolytic diseases. However, the molecular cascade linking *DNL* to STAT3 signaling remains undefined. Key unresolved questions include identifying the specific STAT3 cysteine residue(s) palmitoylated during osteoclastogenesis and elucidating how this modification regulates STAT3 phosphorylation and its transcriptional control of osteoclastogenic genes. Furthermore, whether additional palmitoylation substrates mediate the effect of FASN on osteoclastogenesis remains to be determined. The disparate outcomes of FASN inhibition across studies, ranging from indirect RANKL suppression to direct inhibition of osteoclast differentiation, likely reflect compound-specific off-target effects and different cellular contexts, highlighting the need for head-to-head comparisons of various FASN inhibitors. Lastly, to validate the specific role of *DNL* in regulating osteoclastogenesis, genetic studies using mice with osteoclast precursor-specific knockout of *Fasn* or other components of the *DNL* pathway are needed.

### 3.3 FAO in Osteoclastogenesis

Mitochondrial FAO has emerged as a critical yet paradoxically interpreted metabolic pathway in osteoclast differentiation. Initial evidence from Kushwaha et al. showed that long−chain FAO is dynamically upregulated during RANKL−induced osteoclastogenesis *in vitro (*9). Using a myeloid−specific *Cpt2* knockout mouse model, they demonstrated that genetic inhibition of FAO leads to a pronounced, sex−dependent increase in trabecular bone mass in growing female mice, attributed to fewer and smaller osteoclasts, while males remained largely unaffected (9). Bone marrow cultures from *Cpt2*−deficient mice exhibited impaired formation of large multinucleated osteoclasts, accompanied by features of an intrinsic energetic crisis, including cellular ATP depletion, AMPK activation, and mTOR inhibition. Notably, this defect could not be rescued by excess glucose or pyruvate but was partially restored by the medium−chain FA octanoate, which bypasses CPT2 through direct mitochondrial uptake. Together, these findings established that mitochondrial long−chain FAO serves as a non−redundant energy source required for efficient osteoclastogenesis and normal postnatal bone remodeling, particularly in females.

Huang et al. explored the role of FAO in osteoclastogenesis within a pathological context (15). They reported that FAO and its rate-limiting enzyme CPT1A are significantly upregulated in circulating CD14^+^ monocytes (osteoclast precursors) from RA patients compared to healthy controls. Elevated CPT1A expression positively correlated with increased osteoclast multinucleation and higher radiographic scores of bone erosion. Mechanistically, CPT1A-mediated FAO upregulated the transcription factor C/EBPβ, which directly bound to the promoters of clathrin light chain A (*CLTA*) and clathrin heavy chain (*CLTC*), enhancing their transcription. This increased clathrin expression drives clathrin-dependent endocytosis, leading to accumulation of fusogenic proteins at the plasma membrane and podosome, thereby facilitating osteoclast precursor fusion. Pharmacologic inhibition of CPT1A with etomoxir or genetic knockdown via *siRNA* suppressed osteoclast fusion, suggesting that targeting the CPT1A/FAO axis in peripheral monocytes may represent a therapeutic strategy to inhibit pathological osteoclast precursor fusion and mitigate bone destruction in RA. Nevertheless, how CPT1A-mediated FAO regulates C/EBPβ expression remains to be elucidated.

In stark contrast, a recent study by Chen and colleagues introduced an additional layer of complexity by identifying protein arginine methyltransferase 6 (PRMT6) as an epigenetic suppressor of FAO during early osteoclastogenesis (57). Using multi-omics analyses, they demonstrated that RANKL stimulation upregulates PRMT6, which catalyzes asymmetric dimethylation of histone H3 at arginine 2 (H3R2me2a) at the promoters of key FAO genes, including *Ppard*, *Cpt1a*, and *Acox3*. This repressive histone modification reduces chromatin accessibility, thereby suppressing FAO gene expression. Consequently, PRMT6-mediated FAO repression alleviates the inherent suppressive effect of FAO on glycolysis, while simultaneously promoting HIF-1α-dependent glycolysis, therefore collectively driving a metabolic switch from FAO to glycolysis that is essential for early osteoclast differentiation. Genetic deletion or pharmacological inhibition of PRMT6 reversed this switch, markedly enhancing FAO while impairing osteoclast formation and bone resorption both *in vitro* and in ovariectomy-induced osteoporosis models.

The contrasting findings described above reveal several critical discrepancies. First, while Kushwaha and Huang demonstrate that FAO is required for osteoclast differentiation and fusion, Chen et al. show that FAO must be suppressed to enable the glycolytic shift necessary for early osteoclastogenesis. These discrepancies likely stem from the following four factors: distinct temporal windows (early commitment versus late fusion), unexamined sex dimorphism, the physiological versus pathological context, and different methodological approaches (genetic ablation, pharmacological inhibition, epigenetic manipulation). Regarding temporal dynamics, it is plausible that FAO is initially suppressed to allow glycolytic activation, but subsequently upregulated to meet the energetic demands of later stages of osteoclast fusion and maturation. Direct time-course studies measuring FAO activity, glycolysis dynamics, gene expression, and oxygen consumption rates across defined differentiation stages are urgently needed. Second, striking sex dimorphism was reported by Kushwaha et al., whereas Chen et al. did not stratify their analyses by sex, and Huang et al. studied RA patients of both sexes without reporting sex-dependent differences. Whether PRMT6-mediated FAO suppression exhibits sex-dependent effects, given that PRMT6 was upregulated in estrogen-deficient ovariectomized mice, remains entirely unexplored. Further studies are needed to elucidate the molecular basis of this sex-specific role of FAO in regulating osteoclastogenesis. Third, the context−dependency of FAO requirements is unclear. Huang et al. focused on a pathological RA setting, where chronic inflammation may alter metabolic dependencies, while Chen et al. found that PRMT6 deficiency did not affect bone mass under non−ovariectomized conditions, suggesting PRMT6’s role is predominantly pathological. Whether the CPT1A/FAO axis described by Huang is similarly indispensable under bone homeostasis remains unknown, as conditional knockout models for *Cpt1a* in osteoclasts have not yet been reported. Fourth, methodological approaches differ substantially among these studies. Kushwaha et al. used genetic *Cpt2* ablation, Huang et al. used pharmacological CPT1A inhibition (etomoxir) and *siRNA* knockdown, and Chen et al. indirectly influenced FAO by manipulating PRMT6-mediated epigenetic repression. These distinct strategies may differentially affect metabolic flux and compensatory responses. Additionally, species differences (murine BMMs versus human CD14^+^ monocytes) may contribute to divergent findings. Clearly, it is critical to generate osteoclast lineage-specific conditional knockout mice for *Cpt1a*, *Prmt6*, and *Cpt2* to dissect cell-autonomous versus systemic effects and to examine sex-specific phenotypes. Integrating these seemingly contradictory findings may ultimately reveal that FAO plays stage−specific and context−dependent roles, offering multiple therapeutic windows for modulating osteoclast activity in bone diseases.

### 3.4 FA storage in osteoclastogenesis

TAG synthesis within osteoclast precursors serves as a critical protective mechanism against FA−induced osteoclastogenesis. Drosatos−Tampakaki and colleagues demonstrated that efficient esterification of FAs into TAGs, catalyzed by diacylglycerol acyltransferase 1 (DGAT1), suppresses osteoclast differentiation (45). In contrast, palmitate (PA, 16:0), a saturated FA abundant in the Western diet, is poorly esterified and instead potently enhances RANKL−stimulated osteoclast differentiation. Even in the absence of RANKL, PA alone suffices to induce osteoclast formation, accompanied by increased tartrate-resistant acid phosphatase (TRAP) activity and cell fusion. Mechanistically, the detrimental effect of PA mainly stems from its ability to upregulate TNFα expression, since neutralizing TNFα antibodies completely abolishes PA−driven osteoclast differentiation. Moreover, PA increases the expression of RANK, rendering osteoclast precursors more responsive to RANKL stimulation. Conversely, oleic acid (OA, 18:1), a monounsaturated FA, is readily incorporated into TAG, leading to intracellular LD accumulation. Importantly, OA counteracts PA−induced osteoclastogenesis when co−administered, an effect attributed to enhanced TAG synthesis that sequesters PA and limits its pro−inflammatory availability.

The protective role of TAG synthesis is further supported by gain− and loss−of−function studies of *Dgat1*. Adenovirus−mediated overexpression of *Dgat1* in RAW 264.7 cells enhances TAG formation and inhibits PA−induced TNFα expression and osteoclastogenesis. Conversely, bone marrow cells from *Dgat1*−deficient mice form larger multinucleated osteoclasts with increased TRAP and TNFα mRNA levels. In line with these cellular findings, *Dgat1* knockout mice exhibit reduced bone mass and elevated osteoclast numbers, indicating that endogenous DGAT1 preserves bone microarchitecture by restraining osteoclast formation. The pathophysiological relevance of these findings is underscored by *in vivo* dietary studies. Mice fed a high−fat diet enriched with PA for 12 weeks gained weight similarly to those fed an OA−enriched diet, but the PA−fed group exhibited significantly greater bone loss. Thus, the type of dietary fat, rather than obesity alone, critically influences skeletal health.

Collectively, these findings establish that PA promotes osteoclastogenesis via TNFα and enhanced RANK signaling, while OA and DGAT1 counteract this process by promoting TAG storage, which likely sequesters PA and limits its pro−inflammatory availability. Thus, the anti−osteoclastogenic effect of OA is largely dependent on PA, and whether OA has a PA−independent role in other pathological conditions remains unclear. Several possibilities may explain the mechanisms underlying the stimulatory effect of OA on incorporation of PA into TAG. First, OA treatment increases mRNA expression of *Dgat1*. Second, OA may initiate the TAG synthesis pathway, increasing the cellular capacity to esterify FAs, including PA. Further studies are needed to rigorously test these possibilities. Nevertheless, targeting DGAT1 activity or increasing dietary intake of monounsaturated FAs (e.g., OA) may represent metabolic strategies to restrain pathological osteoclastogenesis. Furthermore, anti−inflammatory approaches, particularly TNFα inhibition, could mitigate PA−driven osteoclast activation and bone loss in conditions such as obesity, diabetes, or postmenopausal osteoporosis.

FA metabolism does not function in isolation but is tightly integrated with energy-sensing and hypoxic signaling pathways during osteoclastogenesis. As highlighted in Section 3.3, PRMT6-mediated epigenetic repression of FAO genes during early osteoclastogenesis alleviates the intrinsic inhibition of glycolysis by FAO and promotes HIF-1α-dependent glycolytic upregulation, a metabolic switch essential for osteoclast commitment. Conversely, genetic FAO impairment (e.g., *Cpt2* knockout) triggers ATP depletion, AMPK activation, and mTOR inhibition, directly linking defective FAO to suppression of the anabolic mTOR pathway. Moreover, FAO-derived acetyl-CoA and NADH feed into the TCA cycle and oxidative phosphorylation, providing ATP and modulating mitochondrial ROS production, which in turn amplifies NF-κB and MAPK signaling (Sections 3.1, 3.3). Thus, FA metabolism serves as both a fuel source and a signaling regulator. Its stage-specific modulation dynamically interfaces with AMPK/mTOR energy sensing and HIF-1α-driven metabolic reprogramming, collectively fine-tuning osteoclast commitment, fusion, and resorptive capacity.

## 4 Translational potential of targeting FA metabolism in treating bone diseases

The expanding knowledge of FA metabolism in osteoclastogenesis has opened new therapeutic avenues for bone diseases driven by excessive osteoclast activity, such as postmenopausal osteoporosis, rheumatoid arthritis, metastatic bone loss, and peri−implant osteolysis. Targeting FA uptake, *DNL*, FAO, and FA storage has shown promise in preclinical models. However, the majority of mechanistic evidence detailed above derives from murine models and *in vitro* cultures, and direct clinical trials for anti-resorptive efficacy of FA metabolic modulators are currently lacking. The available human data are largely indirect, including correlative studies linking FABP4 and CPT1A to disease severity, as well as safety observations from clinical trials investigating these agents for other indications. These limitations underscore key translational challenges that must be addressed before clinical translation, such as species-specific metabolic differences, systemic off-target toxicities, and the need for bone-targeted delivery strategies to achieve skeletal specificity. Below, we summarize the preclinical and clinical status of key FA metabolic modulators and their translational potential (**Table 2**).

**Table 2:** Inhibitors of FA metabolism and their preclinical and clinical status in osteoclastogenesis.

Target (role in FA metabolism)	Inhibitor	Mechanism (targeted domain/activity)	Anti-osteoclastogenic effect in vitro (cell types)	Anti-osteoclastogenic effect in vivo (mouse models)	Clinical status
FATP2 (FA transport/activation)	Lipofermata	FATP2 inhibitor (Transporter activity)	Yes (Murine BMMs)	Yes (HFD-, OVX- and LPS-induced osteolysis)	Not reported
	CB5	FATP2 inhibitor	Not tested	Not tested	Not reported
FABP4 (FA binding)	BMS309403	FABP4 inhibitor	Yes (Murine BMMs)	Yes (OVX-induced osteolysis)	Not reported
CD36 (FA uptake)	Nasunin	Natural TSP-1 inhibitor	Yes	Yes (Type 2 diabetes−induced osteoporosis)	Not reported
	SSO	Irreversible CD36 inhibitor	Not tested	Not tested	Not reported
	SMS121	CD36 modulator	Not tested	Not tested	Not reported
CPT1/2 (FAO)	Etomoxir	Non-selective irreversible CPT1 inhibitor	Yes (CD14+ monocytes from RA patient)	Not tested	Phase II/III for congestive heart failure (ERGO study); discontinued due to hepatotoxicity
	ST1326	Selective CPT1A inhibitor	Not tested	Not tested	No reported
	Perhexiline	CPT1/2 dual inhibitor	Not tested	Not tested	Previously clinically used for angina, but withdrawn in most countries due to severe adverse effects; Phase II for hypertrophic cardiomyopathy.
ACC (DNL)	Soraphen A	ACC inhibitor (BC domain)	Not tested	Not tested	Not reported
	TOFA	ACC inhibitor	Not tested	Not tested	Not reported
	MK-4074	Liver-targeted pan-ACC inhibitor	Not tested	Not tested	Phase I for NAFLD (discontinued due to hypertriglyceridemia)
	PF-05221304	Pan-ACC inhibitor (CT domain)	Not tested	Not tested	Phase II for NAFLD/NASH
	Firsocostat (ND-630)	Pan-ACC inhibitor (BC domain)	Not tested	Not tested	Phase II for NASH
	ND-646/ND-654	Systemic/liver-optimized Pan-ACC inhibitor	Not tested	Not tested	Not reported
	PF-05175157	Systemic pan-ACC inhibitor	Not tested	Not tested	Phase I; caused reversible thrombocytopenia in humans.
	A-908292	Selective ACC2 inhibitor	Not tested	Not tested	Not reported
	(S)-9c, Olefin derivative-2e	Selective ACC2 inhibitor	Not tested	Not tested	Not reported
	Takeda monocyclic/carboxamide derivatives	Selective ACC1 inhibitor	Not tested	Not tested	Not reported
FASN (DNL)	Cerulenin	Irreversible natural FASN inhibitor (KS domain)	No (RAW264.7 cells/Murine BMMs)	Yes, indirectly via RANKL reduction (OVX−induced osteoporosis)	Not reported
	C75	Synthetic FASN inhibitor (KS, ER, TE domains)	Not tested	No (Healthy mice)	Not reported
	Trans-C75	Enantiomer of C75	Yes (Murine BMMs)	Not tested	Not reported
	Orlistat	Irreversible FASN inhibitor (TE domain)	Not tested	Not tested	Approved for obesity; Phase 2 for type I hyperlipoproteinemia; Phase 2 for lung adenocarcinoma.
	IPI-9119	FASN inhibitor (TE domain)	Not tested	Not tested	Not reported
	ASC40 (TVB-2640)	Reversible FASN inhibitor (KR domain)	Yes (Murine BMMs)	Yes (OVX−induced and titanium nanoparticle−induced osteolysis)	Phase I for metastatic castration-resistant prostate cancer; Phase II/III for NASH; Phase II for HER2-positive metastatic breast cancer; Phase II for astrocytoma; and Phase III for recurrent glioblastoma.
	FT-4101	FASN inhibitor (KR domain)	Not tested	Not tested	Phase I/II for NASH; terminated (reason undisclosed)
	Fasnall	Selective FASN inhibitor (KR, ER, MAT domains)	Not tested	Not tested	Not reported
	GSK2194069, BI 99179, TVB-3166	FASN inhibitor (KR domain)	Not tested	Not tested	Not reported
ZDHHCs (S-Palmitoylation)	2-BP	General ZDHHCs inhibitor	Yes (Murine BMMs)	Yes (OVX−induced osteoporosis)	Not reported

### 4.1 Targeting FA uptake and binding

As described in Section 3.1, FATP2, CD36, and FABP4 play key roles in FA uptake and binding during osteoclast differentiation. Consequently, pharmacological inhibitors targeting these proteins therefore hold therapeutic promise (**Table 2**).

#### 4.1.1 FATP2 inhibition

The selective FATP2 transporter inhibitor Lipofermata has shown consistent anti-osteoclastogenic efficacy across multiple osteolytic models. Systemic administration of Lipofermata protects against bone loss induced by HFD, lipopolysaccharide (LPS), or ovariectomy, without overt toxicity to osteoblasts. From a translational perspective, Lipofermata is particularly attractive for lipid-driven osteolysis (e.g., obesity-associated bone loss) and inflammatory bone erosion. However, recent work indicates that Lipofermata exerts cell-type-dependent immunomodulatory effects, attenuating LPS-induced inflammation in monocytes while potentiating inflammasome activation in macrophages (58). This context dependency must be carefully considered when designing therapeutic regimens. An alternative FATP2 inhibitor CB5 has been validated in metabolic tissues but remains unexplored in bones (59). Thus, assessing its activity in osteoclast precursors represents a critical priority for future studies.

#### 4.1.2 FABP4 inhibition

FABP4 amplifies RANKL signaling by potentiating the Ca^2+^/calcineurin/NFATc1 axis (see Section 3.1). The small-molecule FABP4 inhibitor BMS309403 has advanced the farthest toward bone-directed therapy. While oral BMS309403 modestly improves BMD in ovariectomized mice, a major breakthrough came with bone-targeted nanoparticle delivery (17). Bisphosphonate-conjugated nanoparticles concentrate the drug in the bone marrow, restoring trabecular structure and BMD to levels comparable to alendronate (17). This strategy minimizes systemic exposure and off-target effects. Notably, the REV-ERB agonist SR9009 suppresses osteoclastogenesis partly through FABP4 upregulation (60). Thus, modulating FABP4 expression, rather than directly inhibiting it, could represent an alternative therapeutic approach.

#### 4.1.3 TSP-1/CD36 inhibition

CD36 is dispensable for basal osteoclastogenesis but becomes a critical driver under pathological conditions, particularly type 2 diabetes (T2D)-associated bone loss. In T2D, neutrophil-derived thrombospondin-1 (TSP-1) engages CD36 on osteoclast precursors, triggering a PPARγ-POU2F2-c-FOS cascade that enhances resorption (53). Virtual screening identified the natural compound nasunin as a potent TSP-1 inhibitor, which suppresses the CD36-PPARγ-POU2F2 axis, reduces osteoclast differentiation *in vitro*, and ameliorates T2D-induced osteoporosis *in vivo (*53). This proof-of-concept study opens a new avenue for TSP-1/CD36 pathway blockade as a disease-modifying strategy for diabetic skeletal fragility. In contrast, the classical CD36 inhibitor sulfosuccinimidyl oleate (SSO) is also an irreversible mitochondrial respiratory chain inhibitor (61), making it unsuitable for selective osteoclast targeting due to broad toxicity. SMS121, a novel CD36 inhibitor, reduces acute myeloid leukemia (AML) cell viability by curtailing FA uptake (62). However, its efficacy in osteolytic diseases remains unexplored. Notably, recent evidence also identifies CD11b^+^CD36^+^ cells as bone-anabolic macrophages that protect against age-related bone loss (63), indicating that indiscriminate CD36 blockade might be detrimental. Therefore, context-specific strategies, such as targeting the upstream ligand TSP-1 rather than CD36 itself, may be more promising.

Despite these advances, clinical translation of these inhibitors will require careful evaluation of systemic metabolic effects, as global inhibition of FA uptake or binding could disrupt essential metabolic functions in heart, muscle, and liver. Bone−targeted nanoparticle formulations offer a feasible path to overcome these limitations. Additionally, combining FA uptake inhibitors with standard anti−resorptive agents (e.g., bisphosphonates) might achieve synergistic effects at lower doses, reducing off−target toxicity. Future studies should prioritize conditional knockout mice for *Fatp2*, *Fabp4*, and *Cd36* in osteoclast lineages to definitively establish cell−autonomous roles and to validate the therapeutic window of these inhibitors.

### 4.2 Targeting FAO

Given the divergent findings on FAO’s role in osteoclastogenesis (see Section 3.3), therapeutic targeting of this metabolic pathway must be tailored to specific disease contexts. In the pathological setting of RA, the CPT1A/FAO axis is upregulated and promotes clathrin−mediated osteoclast precursor fusion, thereby driving bone erosion (15). Thus, targeting CPT1A represents a therapeutic opportunity for this disease. Two common classes of CPT1 inhibitors, including glycidic acid analogs and the aminocarnitine derivative, have been developed (34, 35). Etomoxir (ETO), a glycidic acid analog, functions as an irreversible CPT1 inhibitor and has been shown to suppress osteoclast fusion *in vitro*, representing a potential therapeutic agent for mitigating bone destruction in RA. However, ETO is a non−selective inhibitor that targets all CPT1 isoforms and has known off−target effects (34, 64). Critically, ETO has been shown to cause hepatotoxicity in some patients during the clinical trials for congestive heart failure (65). Thus, ETO requires structural refinement to eliminate side effects before being translated into an anti-osteoclastogenic drug. In contrast to ETO, ST1326 is an aminocarnitine derivative that selectively inhibits CPT1A and was originally developed for the treatment of diabetes and ketoacidosis (66), but its potential for treating osteolytic diseases remains to be tested. It should be noted that whether the CPT1A/FAO axis is similarly required for basal bone homeostasis is unknown, as conditional *Cpt1a* knockout in osteoclast precursors has not yet been reported. Therefore, ETO or ST1326 is best viewed as a proof−of−concept tool compound rather than a direct clinical candidate. A critical unanswered question is whether FAO dependency is a universal feature of later stages of osteoclastogenesis or a context-specific vulnerability induced by inflammation or estrogen deficiency, a distinction that will determine the therapeutic window of CPT1 inhibitors. Given that CPT2 is also involved in regulating bone resorption in females, developing CPT1/2 dual inhibitors may be useful to treat female-specific bone diseases, such as PMOP. In this regard, the CPT1/2 inhibitor perhexiline was previously used clinically for treating angina and has emerged as a potential anti-cancer agent, but it was withdrawn from clinical use in most countries due to severe adverse effects (67). Additionally, targeting other enzymes involved in FAO may also be worthy of investigation. However, systemic CPT1A or CPT1/2 inhibition could adversely affect tissues that rely heavily on FAO, such as cardiac and skeletal muscle. Future efforts should focus on developing isoform−selective CPT1A inhibitors and bone−targeted delivery strategies to limit systemic exposure.

### 4.3 Targeting FA Storage

Rather than inhibiting FAO, an alternative metabolic strategy is to enhance the sequestration of pro-osteoclastogenic SFAs, such as PA, into inert TAG droplets, thereby limiting their availability to drive TNFα production and RANKL signaling. DGAT1 catalyzes the final step of TAG synthesis and serves as a protective gatekeeper against lipotoxic osteoclastogenesis (45). Direct pharmacological activation of DGAT1 would therefore represent the most straightforward approach. However, no specific DGAT1 activators have been developed to date. Current drug development efforts have instead focused on DGAT1 inhibitors for the treatment of obesity and dyslipidemia, an approach that would be counterproductive for bone health. Consequently, alternative strategies to achieve functional enhancement of DGAT1 activity are needed. Notably, oleic acid (OA; C18:1), a monounsaturated FA abundant in olive oil, is readily incorporated into TAG and counteracts PA-induced osteoclastogenesis by promoting PA sequestration (45). Increasing dietary intake of OA-rich oils could therefore represent a low-risk, readily implementable nutritional intervention to mitigate diet-driven bone loss in conditions including obesity, metabolic syndrome, and type 2 diabetes. Prospective clinical trials are needed to assess the preventive and therapeutic potential of MUFA−enriched diets against pathological bone loss in these diseases.

### 4.4 Targeting *DNL*

As discussed in Section 3.2, *DNL* is upregulated during RANKL-induced osteoclast differentiation and directly promotes osteoclastogenesis via palmitate supply. The two key enzymes of *DNL*, namely ACC1 and FASN, are therefore druggable targets for anti-resorptive therapy. Below we focus on the pharmacological inhibitors targeting these enzymes (**Table 2**), drawing on preclinical and clinical experience from metabolic diseases and oncology.

#### 4.4.1 ACC inhibition

Mammals express two ACC isoforms, cytosolic ACC1 and mitochondrial ACC2. Both contain biotin carboxylase (BC), biotin-containing carboxyl carrier protein (BCCP), central domain (CD), and carboxyltransferase (CT) domains (26). Despite the well-established role of ACC in FA synthesis, no study has directly tested ACC inhibitors in osteoclastogenesis or osteolytic diseases. However, several potent ACC inhibitors have entered clinical development for non-alcoholic fatty liver disease (NAFLD) or non-alcoholic steatohepatitis (NASH), providing valuable insights into their pharmacology and potential liabilities that may guide future bone-targeted strategies.

The first ACC inhibitors studied were soraphen A and TOFA (5-(tetradecyloxy)-2-furancarboxylic acid) (26). Soraphen A, a natural BC domain inhibitor, disrupts ACC oligomerization and shows anti-tumor activity. TOFA is metabolized to TOFyl-CoA, which inhibits ACC but may also target ATP-citrate lyase (ACLY). Both agents have poor bioavailability and lack the specificity required for clinical development, though they established foundational proof-of-concept for ACC inhibition.

Several liver-targeted pan-ACC inhibitors have advanced to clinical trials. MK-4074 reduced hepatic steatosis but was discontinued due to hypertriglyceridemia (68). Similarly, the CT domain inhibitor PF-05221304 and firsocostat (also known as GS-0976 or ND-630) that targets the BC domain and mimics AMPK phosphorylation to disrupt dimerization were developed (69, 70). Both lowered liver fat, yet consistently increased plasma TAGs, an on-target effect resulting from reduced hepatic polyunsaturated FAs and subsequent SREBP1c activation. ND-630-related compounds, including the broadly tissue-distributed ND-646 and liver-optimized ND-654, have demonstrated antitumor activity (71, 72). Notably, systemic pan-ACC inhibition with PF-05175157 caused reversible thrombocytopenia in humans via impaired megakaryocyte maturation, a species-specific toxicity not observed in rodents (73). This adverse event has been circumvented through liver-targeted delivery strategies (74).

In addition to pan-ACC agents, isozyme-selective inhibitors have been developed. A-908292 is an ACC2-selective inhibitor that lowered muscle malonyl-CoA but caused neurological and cardiovascular side effects (26). (S)-9c and olefin derivative-2e, also ACC2-selective, improved glucose homeostasis in preclinical models (75, 76). In contrast, Takeda’s monocyclic and carboxamide derivatives preferentially target ACC1 (75). However, none of these isozyme-selective agents has advanced to clinical testing.

The divergent metabolic consequences of ACC1 versus ACC2 inhibition have important implications for therapeutic targeting in bone diseases. ACC1 blockade would suppress malonyl-CoA production, potentially phenocopying FASN inhibition and blocking osteoclast differentiation by depriving cells of palmitate required for protein palmitoylation. Conversely, ACC2 inhibition would relieve CPT1 suppression, potentially increasing FAO, a metabolic state that may promote rather than inhibit osteoclastogenesis. Given that global ACC inhibition carries risks of thrombocytopenia and hypertriglyceridemia, and that ACC2 inhibition may paradoxically promote FAO, bone-targeted ACC1-selective inhibitors would be required for anti-resorptive therapy. However, none have been developed or tested in osteolytic models. ACC1 therefore remains a plausible but hypothetical target for osteolytic diseases.

#### 4.4.2 FASN inhibition

FASN is a multifunctional enzyme composed of six catalytic domains, including β-ketoacyl synthase (KS), malonyl/acetyl transferase (MAT), dehydratase (DH), enoyl reductase (ER), β-ketoacyl reductase (KR), and thioesterase (TE), along with an additional acyl carrier protein domain (ACP) (21, 30, 77). A wide array of FASN inhibitors targeting different catalytic domains have been developed for various diseases (26). Several of these inhibitors have been investigated in bone-related contexts, although their mechanisms of action and therapeutic potential vary considerably.

Cerulenin is an irreversible natural FASN inhibitor that binds the KS domain. It has been shown to attenuate ovariectomy-induced bone loss, but its anti-osteoclastogenic effect is mediated by downregulating RANKL expression in the bone microenvironment rather than by directly suppressing osteoclast differentiation (16). However, cerulenin suffers from chemical instability and off−target reactivity, which limit its clinical translation. This has led to the synthesis of C75, which targets the KS, ER, and TE domains of FASN (78, 79). A recent study reported that C75 increases bone mass in normal mice by promoting bone formation rather than by suppressing osteoclastogenesis (56). However, C75 also stimulates CPT1 activity and modulate AMPK activity in neurons (80), and it suppresses appetite and causes profound weight loss in rodents (81). These off-target metabolic and behavioral effects confound interpretation of its skeletal effects, and whether the bone phenotype reflects direct FASN inhibition or secondary metabolic adaptations remains unresolved. Notably, trans-C75, an enantiomer of C75, directly suppresses osteoclast differentiation *in vitro (*10). The mechanistic basis for this enantiomer-specific discrepancy is unknown and requires clarification. Whether either enantiomer could be useful for treating osteolytic bone diseases remains to be determined.

Orlistat, an irreversible TE domain inhibitor approved for obesity management, showed no significant effect on bone mass or density in a one-year clinical study of obese patients (82). These negative results should be interpreted cautiously given the small sample size. IPI-9119, another TE inhibitor with potent antitumor activity in preclinical models (83), has not yet entered clinical trials, and its anti-osteoclastogenic efficacy has not been evaluated.

ASC40 (TVB-2640) is an oral, reversible FASN inhibitor that targets the KR domain and has entered clinical trials for the treatment of NASH and multiple cancers (26, 84). It directly suppresses osteoclastogenesis by blocking STAT3 palmitoylation (see Section 3.2 for mechanistic details). In preclinical studies, ASC40 protected against ovariectomy-induced and titanium nanoparticle-induced bone erosion. Importantly, ASC40 has exhibited a favorable safety profile in in oncology trials, with only manageable side effects (85). But whether this tolerability translates to bone disease scenarios and whether the preclinical anti-resorptive efficacy translates to humans remains untested. Its efficacy in inflammatory osteolysis, such as RA, and its potential synergy with standard anti-resorptives are unknown. Similarly, FT-4101, another KR domain inhibitor that entered phase I/II trials for NASH, was well tolerated, and reduced hepatic steatosis in obese NASH patients (86). Other KR-targeting FASN inhibitors, including Fasnall (which also targets ER and MAT domains), GSK2194069, BI 99179, and TVB-3166, have shown antitumor activity in preclinical models (26, 87–90). But none have been evaluated in osteolytic models.

In summary, *DNL* is largely dispensable in most adult tissues under normal dietary conditions and hyperactive osteoclasts depend on FASN-mediated *DNL*. Therefore, FASN inhibition offers a relatively selective metabolic vulnerability in hyperactive osteoclasts. Future studies should evaluate the efficacy and safety of domain-specific inhibitors in osteolytic disease models and develop bone-targeted delivery systems to minimize off-target effects in other tissues.

### 4.5 Targeting protein S-palmitoylation

*DNL* modulates S-palmitoylation, a lipid modification critical for osteoclastogenesis, through controlling palmitate availability (10, 91). Therefore, rather than solely inhibiting *DNL* enzymes, an alternative therapeutic strategy is to directly target *DNL*-mediated protein S-palmitoylation. Consistent with this concept, we recently showed that 2-bromopalmitate (2-BP), a global inhibitor of palmitoylation, suppresses RANKL-induced osteoclast formation *in vitro* and attenuates ovariectomy-induced bone loss in mice (91). However, 2-BP is a palmitate analogue that irreversibly inhibits all PATs. This broad-spectrum activity, together with its promiscuous inhibition of other metabolic enzymes and its cytotoxicity at moderate concentrations, renders it unsuitable for clinical translation (92). A more refined approach is to develop competitive peptides that selectively block the palmitoylation of specific substrates (93, 94). In this regard, FASN-mediated STAT3 palmitoylation has been implicated in osteoclastogenesis. Consequently, peptide-based inhibitors designed to specifically disrupt STAT3 palmitoylation represent a promising alternative. Such targeted interventions could attenuate osteoclastogenesis and ameliorate bone loss while minimizing the off-target effects associated with global palmitoylation blockade. Rigorous preclinical evaluation of these peptides in osteolytic disease models is warranted.

## 5 Conclusion and perspective

In conclusion, FA metabolism has emerged as a critical regulator of osteoclastogenesis, operating through multiple interconnected pathways including uptake, FAO, storage, *DNL*, and protein S-palmitoylation. These metabolic programs not only satisfy the energetic and biosynthetic demands of osteoclast differentiation and function but also generate metabolic intermediates that directly modulate master transcription factors such as NFATc1 and c-Fos.

Preclinical studies have demonstrated that several therapeutic strategies, including FATP2 inhibition (Lipofermata), FABP4 blockade (BMS309403 with bone-targeted nanoparticle delivery), FASN suppression (ASC40), and DGAT1-mediated FA sequestration, all protect against osteolytic bone loss in preclinical disease models. However, significant challenges remain. The paradoxical roles of FA oxidation necessitate stage-specific and context-dependent therapeutic approaches. The species-specific thrombocytopenia associated with systemic ACC inhibition and the hepatotoxicity of CPT1 inhibitors underscore the necessity of tissue-targeted delivery. Moreover, the bone marrow microenvironment, with its unique lipid-rich niche and adipocyte-osteoclast crosstalk, must be considered in therapeutic design. Despite these challenges, among the FA metabolic targets discussed, FASN inhibition with ASC40 currently holds the strongest preclinical evidence for clinical translation, given its direct anti-osteoclastogenic mechanism, efficacy in multiple osteolytic models, and established safety profile in oncology trials.

Future priorities include generating osteoclast lineage-specific conditional knockout models to establish cell-autonomous metabolic requirements, developing bone-targeted formulations to minimize systemic metabolic disruption, resolving the temporal dynamics of metabolic switching during osteoclast differentiation, and translating preclinical findings through rigorous clinical evaluation in osteolytic diseases. Integrating metabolic targeting with existing anti-resorptive agents may ultimately yield synergistic therapies for osteolytic diseases.

## References

[1] Choi IA Umemoto A Mizuno M Park-Min KH . Bone metabolism - an underappreciated player. NPJ Metab Health Dis. (2024) 2:12. doi: 10.1038/s44324-024-00010-9 40604262 PMC12118745

[2] Veis DJ O'Brien CA . Osteoclasts, master sculptors of bone. Annu Rev Pathol. (2023) 18:257–81. doi: 10.1146/annurev-pathmechdis-031521-040919 36207010

[3] Wu Y Gan D Liu Z Qiu D Tan G Xu Z . Osteocytes: master orchestrators of skeletal homeostasis, remodeling, and osteoporosis pathogenesis. Front Cell Dev Biol. (2025) 13:1670716. doi: 10.3389/fcell.2025.1670716 41081044 PMC12508660

[4] Fan Y Hanai JI Le PT Bi R Maridas D DeMambro V . Parathyroid hormone directs bone marrow mesenchymal cell fate. Cell Metab. (2017) 25:661–72. doi: 10.1016/j.cmet.2017.01.001 28162969 PMC5342925

[5] Li Z Bowers E Zhu J Yu H Hardij J Bagchi DP . Lipolysis of bone marrow adipocytes is required to fuel bone and the marrow niche during energy deficits. Elife. (2022) 11:e78496. doi: 10.7554/elife.78496 35731039 PMC9273217

[6] Xu L Zhu J Rong L Yang H Wang B Lu S . Osteoblast-specific down-regulation of NLRP3 inflammasome by aptamer-functionalized liposome nanoparticles improves bone quality in postmenopausal osteoporosis rats. Theranostics. (2024) 14:3945–62. doi: 10.7150/thno.95423 38994035 PMC11234270

[7] Park-Min KH . Mechanisms involved in normal and pathological osteoclastogenesis. Cell Mol Life Sci. (2018) 75:2519–28. doi: 10.1007/s00018-018-2817-9 29670999 PMC9809143

[8] Li B Lee WC Song C Ye L Abel ED Long F . Both aerobic glycolysis and mitochondrial respiration are required for osteoclast differentiation. FASEB J. (2020) 34:11058–67. doi: 10.1096/fj.202000771r 32627870

[9] Kushwaha P Alekos NS Kim SP Li Z Wolfgang MJ Riddle RC . Mitochondrial fatty acid beta-oxidation is important for normal osteoclast formation in growing female mice. Front Physiol. (2022) 13:997358. doi: 10.3389/fphys.2022.997358 36187756 PMC9515402

[10] Xiu C Zhang L Zhang C Zhang Y Luo X Zhang Z . Pharmacologically targeting fatty acid synthase-mediated de novo lipogenesis alleviates osteolytic bone loss by directly inhibiting osteoclastogenesis through suppression of STAT3 palmitoylation and ROS signaling. Metabolism. (2025) 167:156186. doi: 10.1016/j.metabol.2025.156186 40081616

[11] Bae S Lee MJ Mun SH Giannopoulou EG Yong-Gonzalez V Cross JR . MYC-dependent oxidative metabolism regulates osteoclastogenesis via nuclear receptor ERRalpha. J Clin Invest. (2017) 127:2555–68. doi: 10.1172/jci89935 28530645 PMC5490751

[12] Hu G Yu Y Ren Y Tower RJ Zhang GF Karner CM . Glutaminolysis provides nucleotides and amino acids to regulate osteoclast differentiation in mice. EMBO Rep. (2024) 25:4515–41. doi: 10.1038/s44319-024-00255-x 39271775 PMC11467445

[13] Bertels JC He G Long F . Metabolic reprogramming in skeletal cell differentiation. Bone Res. (2024) 12:57. doi: 10.1038/s41413-024-00374-0 39394187 PMC11470040

[14] Stegen S Moermans K Stockmans I Thienpont B Carmeliet G . The serine synthesis pathway drives osteoclast differentiation through epigenetic regulation of NFATc1 expression. Nat Metab. (2024) 6:141–52. doi: 10.1038/s42255-023-00948-y 38200114 PMC10822776

[15] Huang Z Luo R Yang L Chen H Zhang X Han J . CPT1A-mediated fatty acid oxidation promotes precursor osteoclast fusion in rheumatoid arthritis. Front Immunol. (2022) 13:838664. doi: 10.3389/fimmu.2022.838664 35273614 PMC8902079

[16] Bermeo S Al Saedi A Vidal C Khalil M Pang M Troen BR . Treatment with an inhibitor of fatty acid synthase attenuates bone loss in ovariectomized mice. Bone. (2019) 122:114–22. doi: 10.1016/j.bone.2019.02.017 30779961

[17] Xie Q Du X Liang J Shen Y Ling Y Huang Z . FABP4 inhibition suppresses bone resorption and protects against postmenopausal osteoporosis in ovariectomized mice. Nat Commun. (2025) 16:4437. doi: 10.1038/s41467-025-59719-w 40360512 PMC12075751

[18] Li Z Rosen CJ . The multifaceted roles of bone marrow adipocytes in bone and hematopoietic homeostasis. J Clin Endocrinol Metab. (2023) 108:e1465–e72. doi: 10.1210/clinem/dgad355 37315208 PMC12102716

[19] Rendina-Ruedy E Rosen CJ . Lipids in the bone marrow: an evolving perspective. Cell Metab. (2020) 31:219–31. doi: 10.1016/j.cmet.2019.09.015 31668874 PMC7004849

[20] Samovski D Jacome-Sosa M Abumrad NA . Fatty acid transport and signaling: mechanisms and physiological implications. Annu Rev Physiol. (2023) 85:317–37. doi: 10.1146/annurev-physiol-032122-030352 36347219 PMC13221695

[21] Gusenda C Grininger M . Molecular mechanisms of the mammalian fatty acid cycle. Trends Biochem Sci. (2026) 51:39–50. doi: 10.1016/j.tibs.2025.09.005 41203489

[22] Kong X Tao S Ji Z Li J Li H Jin J . FATP2 regulates osteoclastogenesis by increasing lipid metabolism and ROS production. J Bone Miner Res. (2024) 39:737–52. doi: 10.1093/jbmr/zjae034 38477781

[23] Wang H Wang J Cui H Fan C Xue Y Liu H . Inhibition of fatty acid uptake by TGR5 prevents diabetic cardiomyopathy. Nat Metab. (2024) 6:1161–77. doi: 10.1038/s42255-024-01036-5 38698281 PMC11199146

[24] Watkins PA Ellis JM . Peroxisomal acyl-CoA synthetases. Biochim Biophys Acta. (2012) 1822:1411–20. doi: 10.1016/j.bbadis.2012.02.010 22366061 PMC3382043

[25] Ma Y Nenkov M Chen Y Press AT Kaemmerer E Gassler N . Fatty acid metabolism and acyl-CoA synthetases in the liver-gut axis. World J Hepatol. (2021) 13:1512–33. doi: 10.4254/wjh.v13.i11.1512 PMC8637682 34904027

[26] Batchuluun B Pinkosky SL Steinberg GR . Lipogenesis inhibitors: therapeutic opportunities and challenges. Nat Rev Drug Discov. (2022) 21:283–306. doi: 10.1038/s41573-021-00367-2 35031766 PMC8758994

[27] Beretta M Vancuylenburg CS Shrestha R Olzomer EM Osborne B Zhou M . Isotype-selective roles of hepatic acetyl-CoA carboxylases in a mouse model of fatty liver disease. Mol Metab. (2025) 102:102264. doi: 10.1016/j.molmet.2025.102264 41047119 PMC12555813

[28] McDonald-Hyman C Aguilar EG Compeer EB Zaiken MC Rhee SY Mohamed FA . Acetyl-CoA carboxylase 1 inhibition increases Treg metabolism and graft-versus-host disease treatment efficacy via mitochondrial fusion. J Clin Invest. (2025) 135:e182480. doi: 10.1172/jci182480 41026528 PMC12646659

[29] Savage DB Choi CS Samuel VT Liu ZX Zhang D Wang A . Reversal of diet-induced hepatic steatosis and hepatic insulin resistance by antisense oligonucleotide inhibitors of acetyl-CoA carboxylases 1 and 2. J Clin Invest. (2006) 116:817–24. doi: 10.1172/jci27300 16485039 PMC1366503

[30] Choi W Li C Chen Y Wang Y Cheng Y . Structural dynamics of human fatty acid synthase in the condensing cycle. Nature. (2025) 641:529–36. doi: 10.1038/s41586-025-08782-w 39978408 PMC12058526

[31] Ferrero E Vaz FM Cheillan D Brusco A Marelli C . The ELOVL proteins: very and ultra long-chain fatty acids at the crossroads between metabolic and neurodegenerative disorders. Mol Genet Metab. (2025) 144:109050. doi: 10.1016/j.ymgme.2025.109050 39946831

[32] Kalyesubula M Von Bank H Davidson JW Burhans MS Becker MM Aljohani A . Stearoyl-CoA desaturase 1 deficiency drives saturated lipid accumulation and increases liver and plasma acylcarnitines. J Lipid Res. (2025) 66:100824. doi: 10.1016/j.jlr.2025.100824 40350036 PMC12173144

[33] Zhang L Yao Y Liu S . Targeting fatty acid metabolism for cancer therapy. Fundam Res. (2026) 6:1171–80. doi: 10.1016/j.fmre.2024.09.011 41971814 PMC13069628

[34] McGuffee RM McCommis KS Ford DA . Etomoxir: an old dog with new tricks. J Lipid Res. (2024) 65:100604. doi: 10.1016/j.jlr.2024.100604 39094770 PMC11395757

[35] Schlaepfer IR Joshi M . CPT1A-mediated fat oxidation, mechanisms, and therapeutic potential. Endocrinology. (2020) 161:bqz046. doi: 10.1210/endocr/bqz046 31900483

[36] Guerra IMS Ferreira HB Melo T Rocha H Moreira S Diogo L . Mitochondrial fatty acid beta-oxidation disorders: from disease to lipidomic studies-a critical review. Int J Mol Sci. (2022) 23:13933. doi: 10.3390/ijms232213933 36430419 PMC9696092

[37] Zadoorian A Du X Yang H . Lipid droplet biogenesis and functions in health and disease. Nat Rev Endocrinol. (2023) 19:443–59. doi: 10.1038/s41574-023-00845-0 37221402 PMC10204695

[38] Olzmann JA Carvalho P . Dynamics and functions of lipid droplets. Nat Rev Mol Cell Biol. (2019) 20:137–55. doi: 10.1038/s41580-018-0085-z 30523332 PMC6746329

[39] Mathiowetz AJ Olzmann JA . Lipid droplets and cellular lipid flux. Nat Cell Biol. (2024) 26:331–45. doi: 10.1038/s41556-024-01364-4 38454048 PMC11228001

[40] Qu M Zhou X Wang X Li H . Lipid-induced S-palmitoylation as a vital regulator of cell signaling and disease development. Int J Biol Sci. (2021) 17:4223–37. doi: 10.7150/ijbs.64046 34803494 PMC8579454

[41] Xiu C Ji K Zhao G Chen J Yang Y . The emerging role of palmitoyl acyltransferase zDHHC5 in health and disease: a review. Int J Biol Macromol. (2025) 330:148281. doi: 10.1016/j.ijbiomac.2025.148281 41086885

[42] He Q Qu M Shen T Su J Xu Y Xu C . Control of mitochondria-associated endoplasmic reticulum membranes by protein S-palmitoylation: novel therapeutic targets for neurodegenerative diseases. Ageing Res Rev. (2023) 87:101920. doi: 10.1016/j.arr.2023.101920 37004843

[43] Ji X Xu C Luo J Hu X Wu X . S-palmitoylation in bone biology: emerging insights and therapeutic potential. Biochem Pharmacol. (2025) 242:117232. doi: 10.1016/j.bcp.2025.117232 40848859

[44] Ledesma-Colunga MG Passin V Lademann F Hofbauer LC Rauner M . Novel insights into osteoclast energy metabolism. Curr Osteoporos Rep. (2023) 21:660–69. doi: 10.1007/s11914-023-00825-3 37816910 PMC10724336

[45] Drosatos-Tampakaki Z Drosatos K Siegelin Y Gong S Khan S Van Dyke T . Palmitic acid and DGAT1 deficiency enhance osteoclastogenesis, while oleic acid-induced triglyceride formation prevents it. J Bone Miner Res. (2014) 29:1183–95. doi: 10.1002/jbmr.2150 24272998 PMC4945760

[46] Oh SR Sul OJ Kim YY Kim HJ Yu R Suh JH . Saturated fatty acids enhance osteoclast survival. J Lipid Res. (2010) 51:892–9. doi: 10.1194/jlr.m800626 20388920 PMC2853456

[47] van Heerden B Kasonga A Kruger MC Coetzee M . Palmitoleic acid inhibits RANKL-induced osteoclastogenesis and bone resorption by suppressing NF-kappaB and MAPK signalling pathways. Nutrients. (2017) 9:441. doi: 10.3390/nu9050441 28452958 PMC5452171

[48] Sun D Krishnan A Zaman K Lawrence R Bhattacharya A Fernandes G . Dietary n-3 fatty acids decrease osteoclastogenesis and loss of bone mass in ovariectomized mice. J Bone Miner Res. (2003) 18:1206–16. doi: 10.1359/jbmr.2003.18.7.1206 12854830

[49] Lucas S Omata Y Hofmann J Bottcher M Iljazovic A Sarter K . Short-chain fatty acids regulate systemic bone mass and protect from pathological bone loss. Nat Commun. (2018) 9:55. doi: 10.1038/s41467-017-02490-4 29302038 PMC5754356

[50] Helming L Winter J Gordon S . The scavenger receptor CD36 plays a role in cytokine-induced macrophage fusion. J Cell Sci. (2009) 122:453–9. doi: 10.1242/jcs.037200 19155290 PMC2714432

[51] Koduru SV Sun BH Walker JM Zhu M Simpson C Dhodapkar M . The contribution of cross-talk between the cell-surface proteins CD36 and CD47-TSP-1 in osteoclast formation and function. J Biol Chem. (2018) 293:15055–69. doi: 10.1074/jbc.ra117.000633 30082316 PMC6166722

[52] Carron JA Wagstaff SC Gallagher JA Bowler WB . A CD36-binding peptide from thrombospondin-1 can stimulate resorption by osteoclasts *in vitro*. Biochem Biophys Res Commun. (2000) 270:1124–7. doi: 10.1006/bbrc.2000.2574 10772961

[53] Huang W Zhou C Wu R Zhao K Wu J Song D . Neutrophil-derived thrombospondin-1 (THBS1) drives type 2 diabetes-induced osteoporosis via CD36-PPARgamma-POU2F2 signaling. Biochim Biophys Acta Mol Basis Dis. (2026) 1872:168139. doi: 10.1016/j.bbadis.2025.168139 41407214

[54] Pan C Li S Li C Ko WC Liu T Liu H . Fabkin promoted osteoclasts mature and bone loss in OVX-induced osteoporosis mice. FASEB J. (2025) 39:e71028. doi: 10.2139/ssrn.5254757 40965974

[55] Guo D Lin C Lu Y Guan H Qi W Zhang H . FABP4 secreted by M1-polarized macrophages promotes synovitis and angiogenesis to exacerbate rheumatoid arthritis. Bone Res. (2022) 10:45. doi: 10.1038/s41413-022-00211-2 35729106 PMC9213409

[56] Tang W Ding Z Gao H Yan Q Liu J Han Y . Targeting Kindlin-2 in adipocytes increases bone mass through inhibiting FAS/PPARgamma/FABP4 signaling in mice. Acta Pharm Sin B. (2023) 13:4535–52. doi: 10.1016/j.apsb.2023.07.001 37969743 PMC10638509

[57] Chu W Peng W Lu Y Liu Y Li Q Wang H . PRMT6 epigenetically drives metabolic switch from fatty acid oxidation toward glycolysis and promotes osteoclast differentiation during osteoporosis. Adv Sci (Weinh). (2024) 11:e2403177. doi: 10.1002/advs.202403177 39120025 PMC11516099

[58] Hahn N Chornyi S Heister D de Vries HE Giera M Boon MR . Fatty acid binding protein 2 (FATP2/SLC27A2) blockade with Lipofermata elicits dual effects on inflammatory responses in human monocytes and macrophages. Immunol Lett. (2026) 277:107092. doi: 10.1016/j.imlet.2025.107092 41015393

[59] Saini N Black PN Montefusco D DiRusso CC . Fatty acid transport protein-2 inhibitor Grassofermata/CB5 protects cells against lipid accumulation and toxicity. Biochem Biophys Res Commun. (2015) 465:534–41. doi: 10.1016/j.bbrc.2015.08.055 26284975 PMC4589248

[60] Song C Tan P Zhang Z Wu W Dong Y Zhao L . REV-ERB agonism suppresses osteoclastogenesis and prevents ovariectomy-induced bone loss partially via FABP4 upregulation. FASEB J. (2018) 32:3215–28. doi: 10.1096/fj.201600825rrr 29401617

[61] Drahota Z Vrbacky M Nuskova H Kazdova L Zidek V Landa V . Succinimidyl oleate, established inhibitor of CD36/FAT translocase inhibits complex III of mitochondrial respiratory chain. Biochem Biophys Res Commun. (2010) 391:1348–51. doi: 10.1016/j.bbrc.2009.12.050 20006584

[62] Abacka H Masoni S Poli G Huang P Gusso F Granchi C . SMS121, a new inhibitor of CD36, impairs fatty acid uptake and viability of acute myeloid leukemia. Sci Rep. (2024) 14:9104. doi: 10.1038/s41598-024-58689-1 38643249 PMC11032350

[63] Koroth J Karkache IY Vu EK Mansky KC Bradley EW . CD11B+CD36+ cells are bone anabolic macrophages that limit age-associated bone loss. bioRxiv. (2024). doi: 10.1101/2024.09.13.612932

[64] Moon SH Liu X Jenkins CM Dilthey BG Patti GJ Gross RW . Etomoxir-carnitine, a novel pharmaco-metabolite of etomoxir, inhibits phospholipases A(2) and mitochondrial respiration. J Lipid Res. (2024) 65:100611. doi: 10.1016/j.jlr.2024.100611 39094773 PMC11402452

[65] Holubarsch CJ Rohrbach M Karrasch M Boehm E Polonski L Ponikowski P . A double-blind randomized multicentre clinical trial to evaluate the efficacy and safety of two doses of etomoxir in comparison with placebo in patients with moderate congestive heart failure: the ERGO (etomoxir for the recovery of glucose oxidation) study. Clin Sci (Lond). (2007) 113:205–12. doi: 10.1042/cs20060307 17319797

[66] Giannessi F Pessotto P Tassoni E Chiodi P Conti R De Angelis F . Discovery of a long-chain carbamoyl aminocarnitine derivative, a reversible carnitine palmitoyltransferase inhibitor with antiketotic and antidiabetic activity. J Med Chem. (2003) 46:303–9. doi: 10.1021/jm020979u 12519067

[67] Dhakal B Tomita Y Drew P Price T Maddern G Smith E . Perhexiline: Old drug, new tricks? A summary of its anti-cancer effects. Molecules. (2023) 28:3624. doi: 10.3390/molecules28083624 37110858 PMC10145508

[68] Kim CW Addy C Kusunoki J Anderson NN Deja S Fu X . Acetyl CoA carboxylase inhibition reduces hepatic steatosis but elevates plasma triglycerides in mice and humans: A bedside to bench investigation. Cell Metab. (2017) 26:394–406:e6. doi: 10.1016/j.cmet.2017.07.009 28768177 PMC5603267

[69] Bergman A Carvajal-Gonzalez S Tarabar S Saxena AR Esler WP Amin NB . Safety, tolerability, pharmacokinetics, and pharmacodynamics of a liver-targeting acetyl-CoA carboxylase inhibitor (PF-05221304): A three-part randomized phase 1 study. Clin Pharmacol Drug Dev. (2020) 9:514–26. doi: 10.1002/cpdd.782 32065514 PMC7317421

[70] Harriman G Greenwood J Bhat S Huang X Wang R Paul D . Acetyl-CoA carboxylase inhibition by ND-630 reduces hepatic steatosis, improves insulin sensitivity, and modulates dyslipidemia in rats. Proc Natl Acad Sci USA. (2016) 113:E1796–805. doi: 10.1073/pnas.1520686113 26976583 PMC4822632

[71] Lally JSV Ghoshal S DePeralta DK Moaven O Wei L Masia R . Inhibition of acetyl-CoA carboxylase by phosphorylation or the inhibitor ND-654 suppresses lipogenesis and hepatocellular carcinoma. Cell Metab. (2019) 29:174–82:e5. doi: 10.1016/j.cmet.2018.08.020 30244972 PMC6643297

[72] Svensson RU Parker SJ Eichner LJ Kolar MJ Wallace M Brun SN . Inhibition of acetyl-CoA carboxylase suppresses fatty acid synthesis and tumor growth of non-small-cell lung cancer in preclinical models. Nat Med. (2016) 22:1108–19. doi: 10.1038/nm.4181 27643638 PMC5053891

[73] Kelly KL Reagan WJ Sonnenberg GE Clasquin M Hales K Asano S . De novo lipogenesis is essential for platelet production in humans. Nat Metab. (2020) 2:1163–78. doi: 10.1038/s42255-020-00272-9 32929234

[74] Huard K Smith AC Cappon G Dow RL Edmonds DJ El-Kattan A . Optimizing the benefit/risk of acetyl-CoA carboxylase inhibitors through liver targeting. J Med Chem. (2020) 63:10879–96. doi: 10.1021/acs.jmedchem.0c00640 32809824

[75] Mizojiri R Nii N Asano M Sasaki M Satoh Y Yamamoto Y . Design and synthesis of a novel 1H-pyrrolo[3,2-b]pyridine-3-carboxamide derivative as an orally available ACC1 inhibitor. Bioorg Med Chem. (2019) 27:2521–30. doi: 10.1016/j.bmc.2019.03.023 30879862

[76] Nishiura Y Matsumura A Kobayashi N Shimazaki A Sakamoto S Kitade N . Discovery of a novel olefin derivative as a highly potent and selective acetyl-CoA carboxylase 2 inhibitor with *in vivo* efficacy. Bioorg Med Chem Lett. (2018) 28:2498–503. doi: 10.1016/j.bmcl.2018.05.055 29903660

[77] Schultz K Costa-Pinheiro P Gardner L Pinheiro LV Ramirez-Solis J Gardner SM . Snapshots of acyl carrier protein shuttling in human fatty acid synthase. Nature. (2025) 641:520–8. doi: 10.1038/s41586-025-08587-x 39979457 PMC12058525

[78] Kuhajda FP Pizer ES Li JN Mani NS Frehywot GL Townsend CA . Synthesis and antitumor activity of an inhibitor of fatty acid synthase. Proc Natl Acad Sci USA. (2000) 97:3450–4. doi: 10.1073/pnas.97.7.3450 10716717 PMC16260

[79] Rendina AR Cheng D . Characterization of the inactivation of rat fatty acid synthase by C75: inhibition of partial reactions and protection by substrates. Biochem J. (2005) 388:895–903. doi: 10.1042/bj20041963 15715522 PMC1183470

[80] Landree LE Hanlon AL Strong DW Rumbaugh G Miller IM Thupari JN . C75, a fatty acid synthase inhibitor, modulates AMP-activated protein kinase to alter neuronal energy metabolism. J Biol Chem. (2004) 279:3817–27. doi: 10.1074/jbc.m310991200 14615481

[81] Hu Z Cha SH van Haasteren G Wang J Lane MD . Effect of centrally administered C75, a fatty acid synthase inhibitor, on ghrelin secretion and its downstream effects. Proc Natl Acad Sci USA. (2005) 102:3972–7. doi: 10.1073/pnas.0500619102 15728730 PMC549511

[82] Gotfredsen A Westergren Hendel H Andersen T . Influence of orlistat on bone turnover and body composition. Int J Obes Relat Metab Disord. (2001) 25:1154–60. doi: 10.1038/sj.ijo.0801639 11486790

[83] Zadra G Ribeiro CF Chetta P Ho Y Cacciatore S Gao X . Inhibition of de novo lipogenesis targets androgen receptor signaling in castration-resistant prostate cancer. Proc Natl Acad Sci USA. (2019) 116:631–40. doi: 10.1073/pnas.1808834116 30578319 PMC6329966

[84] Vanauberg D Schulz C Lefebvre T . Involvement of the pro-oncogenic enzyme fatty acid synthase in the hallmarks of cancer: a promising target in anti-cancer therapies. Oncogenesis. (2023) 12:16. doi: 10.1038/s41389-023-00460-8 36934087 PMC10024702

[85] Falchook G Infante J Arkenau HT Patel MR Dean E Borazanci E . First-in-human study of the safety, pharmacokinetics, and pharmacodynamics of first-in-class fatty acid synthase inhibitor TVB-2640 alone and with a taxane in advanced tumors. EClinicalMedicine. (2021) 34:100797. doi: 10.1016/j.eclinm.2021.100797 33870151 PMC8040281

[86] Beysen C Schroeder P Wu E Brevard J Ribadeneira M Lu W . Inhibition of fatty acid synthase with FT-4101 safely reduces hepatic de novo lipogenesis and steatosis in obese subjects with non-alcoholic fatty liver disease: Results from two early-phase randomized trials. Diabetes Obes Metab. (2021) 23:700–10. doi: 10.1111/dom.14272 33289350 PMC7898808

[87] Alwarawrah Y Hughes P Loiselle D Carlson DA Darr DB Jordan JL . Fasnall, a selective FASN inhibitor, shows potent anti-tumor activity in the MMTV-Neu model of HER2(+) breast cancer. Cell Chem Biol. (2016) 23:678–88. doi: 10.1016/j.chembiol.2016.04.011 27265747 PMC6443244

[88] Hardwicke MA Rendina AR Williams SP Moore ML Wang L Krueger JA . A human fatty acid synthase inhibitor binds beta-ketoacyl reductase in the keto-substrate site. Nat Chem Biol. (2014) 10:774–9. doi: 10.1038/nchembio.1603 25086508

[89] Singha PK Maklin K Vihavainen T Laitinen T Nevalainen TJ Patil MR . Evaluation of FASN inhibitors by a versatile toolkit reveals differences in pharmacology between human and rodent FASN preparations and in antiproliferative efficacy *in vitro* vs. in situ in human cancer cells. Eur J Pharm Sci. (2020) 149:105321. doi: 10.1016/j.ejps.2020.105321 32275951

[90] Wang Y Chen Z Li R Wei D Wang S Luo H . S-palmitoylation of c-MET by CK2alpha-mediated zDHHC15 phosphorylation drives glioblastoma stem cell tumorigenicity. Neuro Oncol. (2025) 27:1972–86. doi: 10.1093/neuonc/noaf098 40207776 PMC12448808

[91] Ma L Zhang L Liao Z Xiu C Luo X Luo N . Pharmacological inhibition of protein S-palmitoylation suppresses osteoclastogenesis and ameliorates ovariectomy-induced bone loss. J Orthop Translat. (2023) 42:1–14. doi: 10.1016/j.jot.2023.06.002 37521493 PMC10372326

[92] Ma Y Chen Y Li L Wu Z Cao H Zhu C . 2-Bromopalmitate-induced intestinal flora changes and testicular dysfunction in mice. Int J Mol Sci. (2024) 25:11415. doi: 10.3390/ijms252111415 39518967 PMC11547043

[93] Yao H Lan J Li C Shi H Brosseau JP Wang H . Inhibiting PD-L1 palmitoylation enhances T-cell immune responses against tumours. Nat BioMed Eng. (2019) 3:306–17. doi: 10.1038/s41551-019-0375-6 30952982

[94] Zhang H Sun Y Wang Z Huang X Tang L Jiang K . ZDHHC20-mediated S-palmitoylation of YTHDF3 stabilizes MYC mRNA to promote pancreatic cancer progression. Nat Commun. (2024) 15:4642. doi: 10.1038/s41467-024-49105-3 38821916 PMC11143236

[95] Jiang S Li H Zhang L Mu W Zhang Y Chen T . Generic Diagramming Platform (GDP): a comprehensive database of high-quality biomedical graphics. Nucleic Acids Res. (2025) 53:D1670–6. doi: 10.1093/nar/gkae973 39470721 PMC11701665

